# Remedy for Favus

**Published:** 1854-08

**Authors:** 


*Remedy for Favus.*—From the observation of about a dozen cases of severe favus (diagnosis by the microscope in all) recently treated by Mr. Startin at the Hospital for Skin Diseases, we can speak with great confidence of the efficiency of the following ointment. It is the Ung. sulph. comp. of the Pharmacopœia of that Institution.

℞ Sulph. sublimati lbss.;

Hydrarg. ammoniochloride. ℥ss.;

Hydr. sulphureti cum sulph. ℥ss.

Leviga simul, dein adde olivæ olei ℥iv.;

Adipis recentis ℥xvj.;

Creosoti ♏xx;

Misce.

To correct the state of general health, Mr. Startin commonly orders simultaneously a mild course of the iodide of potassium, but this, we suspect, has but a small share, if any, in the local result. Often when the scalp has been for many years thickly covered with the peculiar favus crust, four or five nightly applications of the above ointment have sufficed to make it perfectly clean. So long as the patient will continue regularly to use a small quantity every day, the disease may be prevented from reappearing, and the condition assumed by the scalp under its influence might easily be mistaken by the inexperienced for one of complete cure. As soon, however, as the inunction is suspended, the eruption reappears. This liability we have known, in more than one case, to extend over nearly a year, and probably it may for much longer periods. The ointment, however, which does not smell much, need only be applied at night, and may be washed entirely away every morning, so as to entail but little inconvenience on the patient. The hair will, to a considerable extent, grow during the treatment, provided that the scalp have not been too much destroyed. In a most disgusting disease, for which as yet no real cure is known, it is much to be in possession of an almost certain means of ensuring its absence. The ointment no doubt acts as a parasiticide. Before its first application it is desirable to clear away the crust as much as possible, either by fomentation or a poultice.

We may remark, that the ointment mentioned is used by Mr. Startin in the treatment of scabies, and also in that of the contagious form of porrigo. —*London Med. Times.*

